# Supplementary figures and images for: A redox-active crosslinker reveals an essential and inhibitable oxidative folding network in the endoplasmic reticulum of malaria parasites
Source: PLoS Pathog. 2021 Feb 3;17(2):e1009293. doi: 10.1371/journal.ppat.1009293 (PMC7886143; doi:10.1371/journal.ppat.1009293)

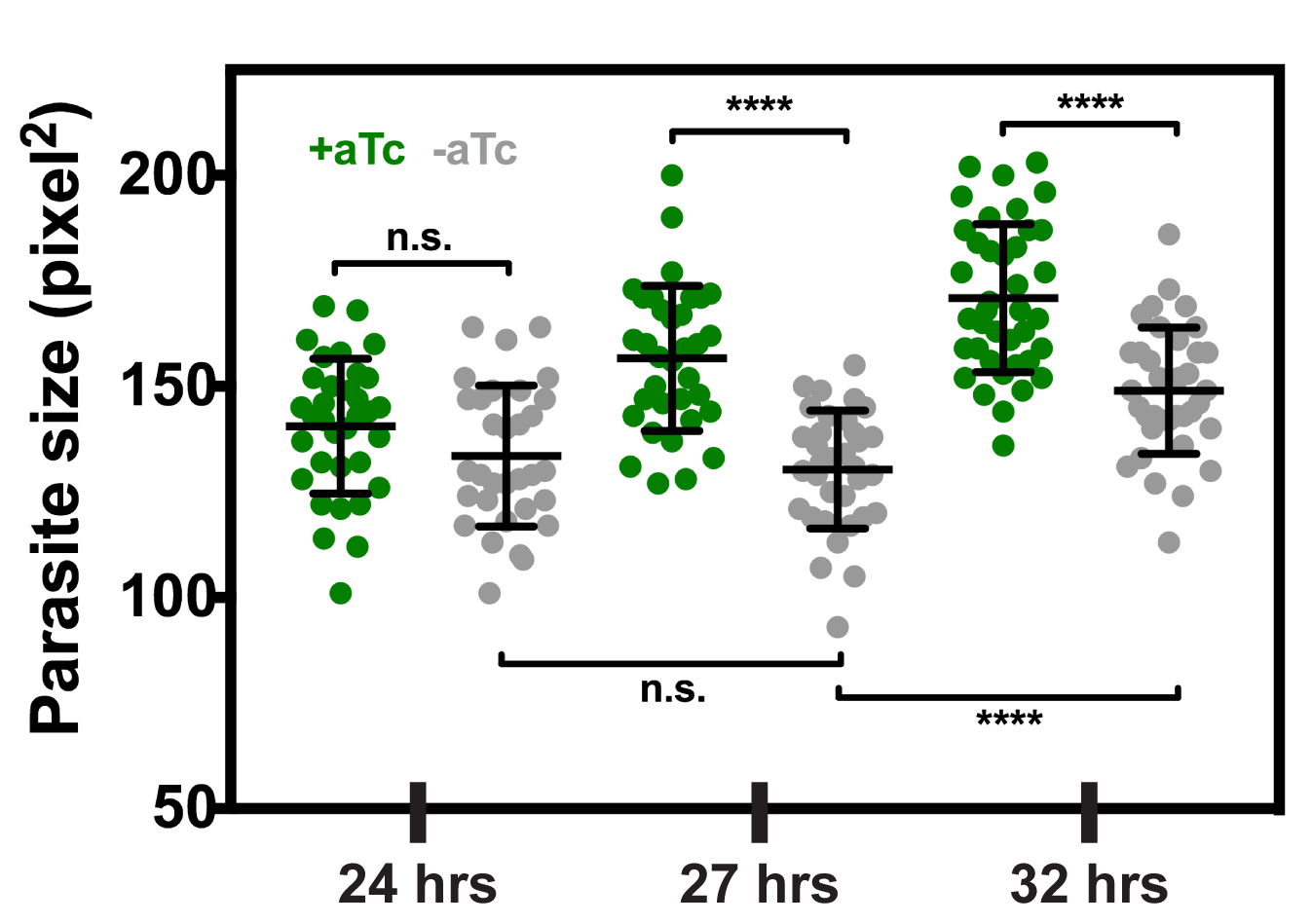

Supplement: S1 Fig — PfJ2apt parasites were tightly synchronized (0–3 hours) to the ring stage, then split into either +aTc (10 nM) or–aTc medium. Smears were made and field-stained at various time points throughout the asexual lifecycle. Stained slides were imaged and parasite size was measured. Unpaired t-test, **** indicates p ≤ 0.0001. Representative experiment of 3 biological replicates shown. (TIF) [file ppat.1009293.s005.tif]

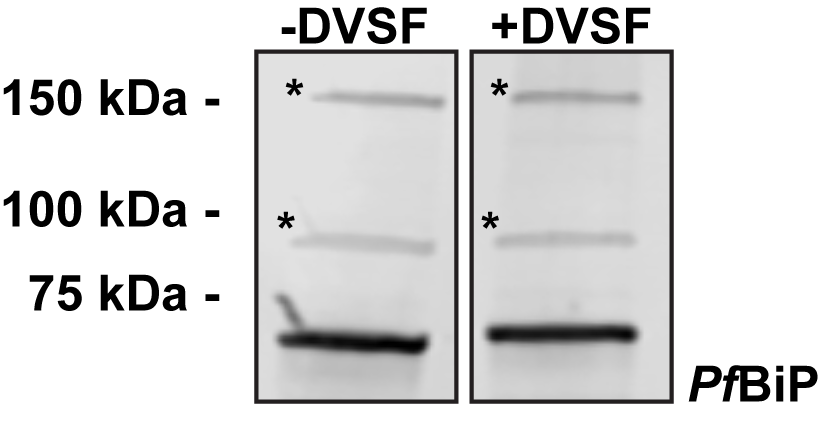

Supplement: S2 Fig — PfJ2apt-PDI8glms parasites were treated with 3 mM DVSF in 1xPBS for 30 minutes at 37°C, or left untreated as a control, and parasite lysates were used for western blotting. Membranes were probed with antibodies against PfBiP. Asterisks (*) denote nonspecific bands. (TIF) [file ppat.1009293.s006.tif]

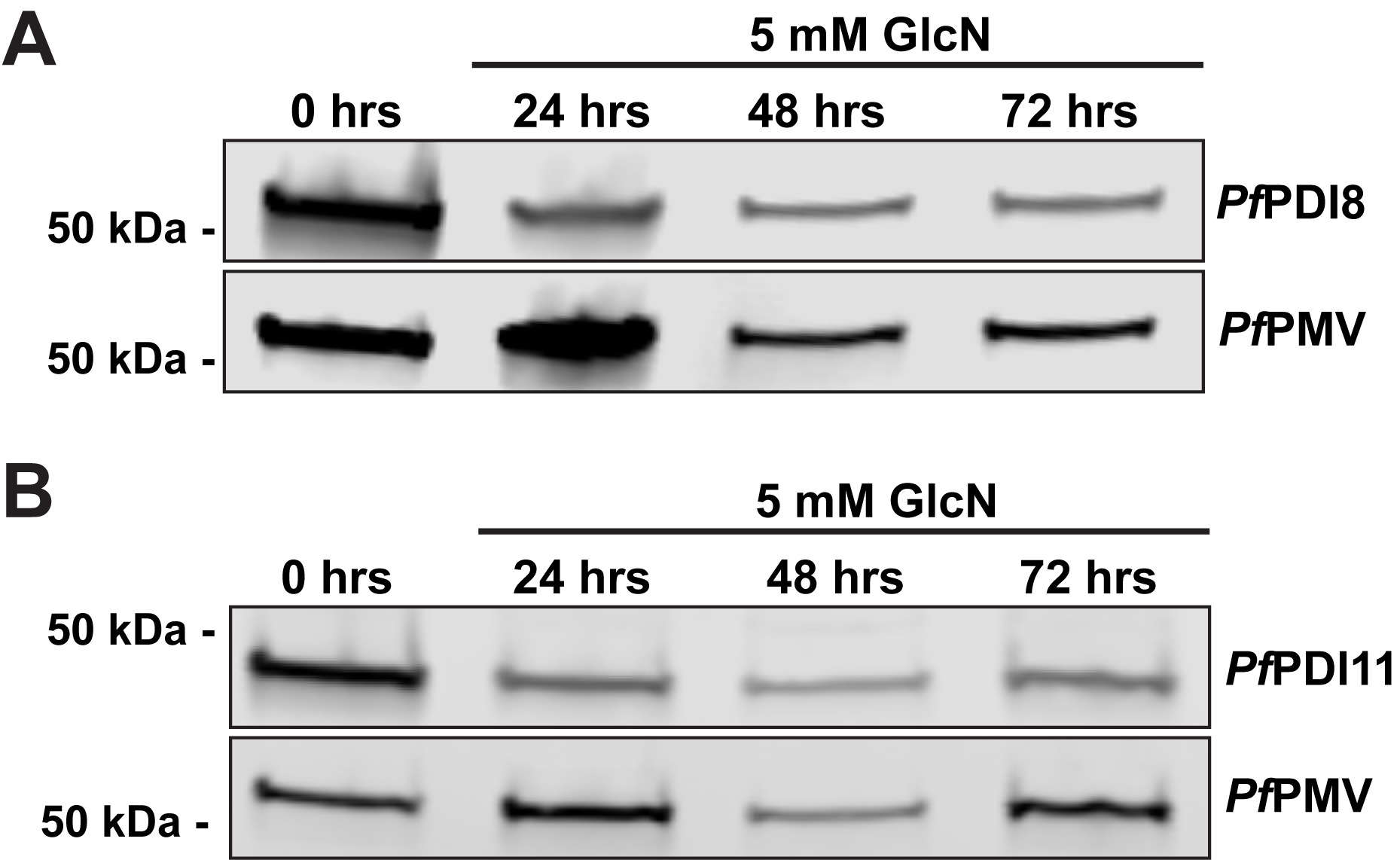

Supplement: S3 Fig — Asynchronous PfJ2apt-PDI8glms (top) and PfJ2apt-PDI11glms (bottom) parasites were treated with 5 mM GlcN and samples were taken for western blot analysis at 0 (before addition of GlcN), 24, 48, and 72 hours. Membranes were probed with antibodies for V5 and PfPMV. (TIF) [file ppat.1009293.s007.tif]

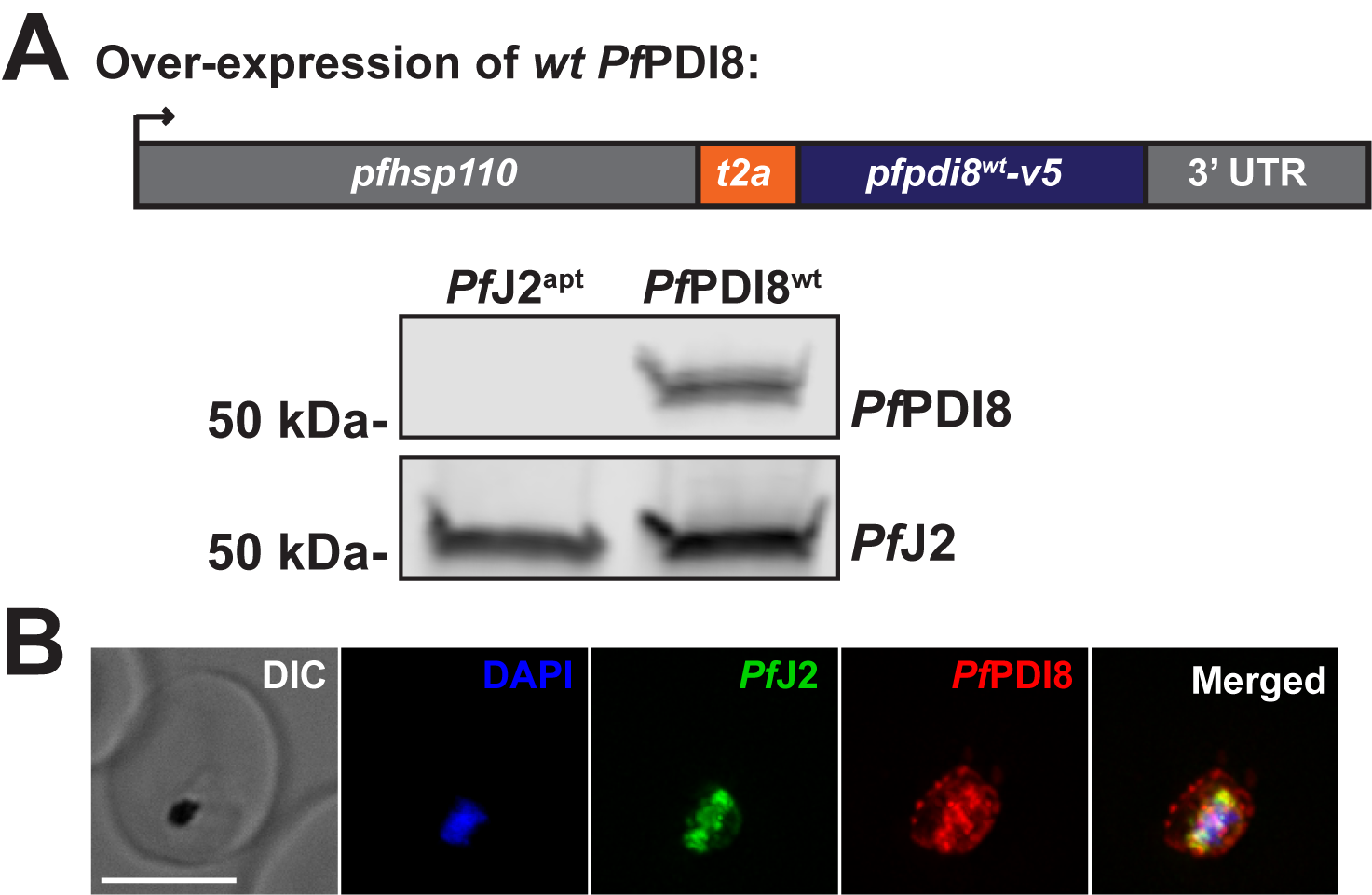

Supplement: S4 Fig — A) Top: schematic of exogenous V5-tagged, wild-type PfPDI8 expression using the pfhsp110 (PF3D7_ 0708800) locus and a T2A skip peptide in the PfPDI8wt parasite line, created in the background of PfJ2apt parasites. Bottom: western blot of parental PfJ2apt and PfPDI8wt parasite lysates, probed for antibodies against the V5 tag (PfPDI8) and the HA tag (PfJ2). B) PfPDI8wt parasites were glutaraldehyde/paraformaldehyde fixed and used for IFA. Staining was carried out using DAPI (blue), antibodies against the HA tag (PfJ2, green), and the V5 tag (PfPDI8, red). Scale bar represents 5 μm. (TIF) [file ppat.1009293.s008.tif]

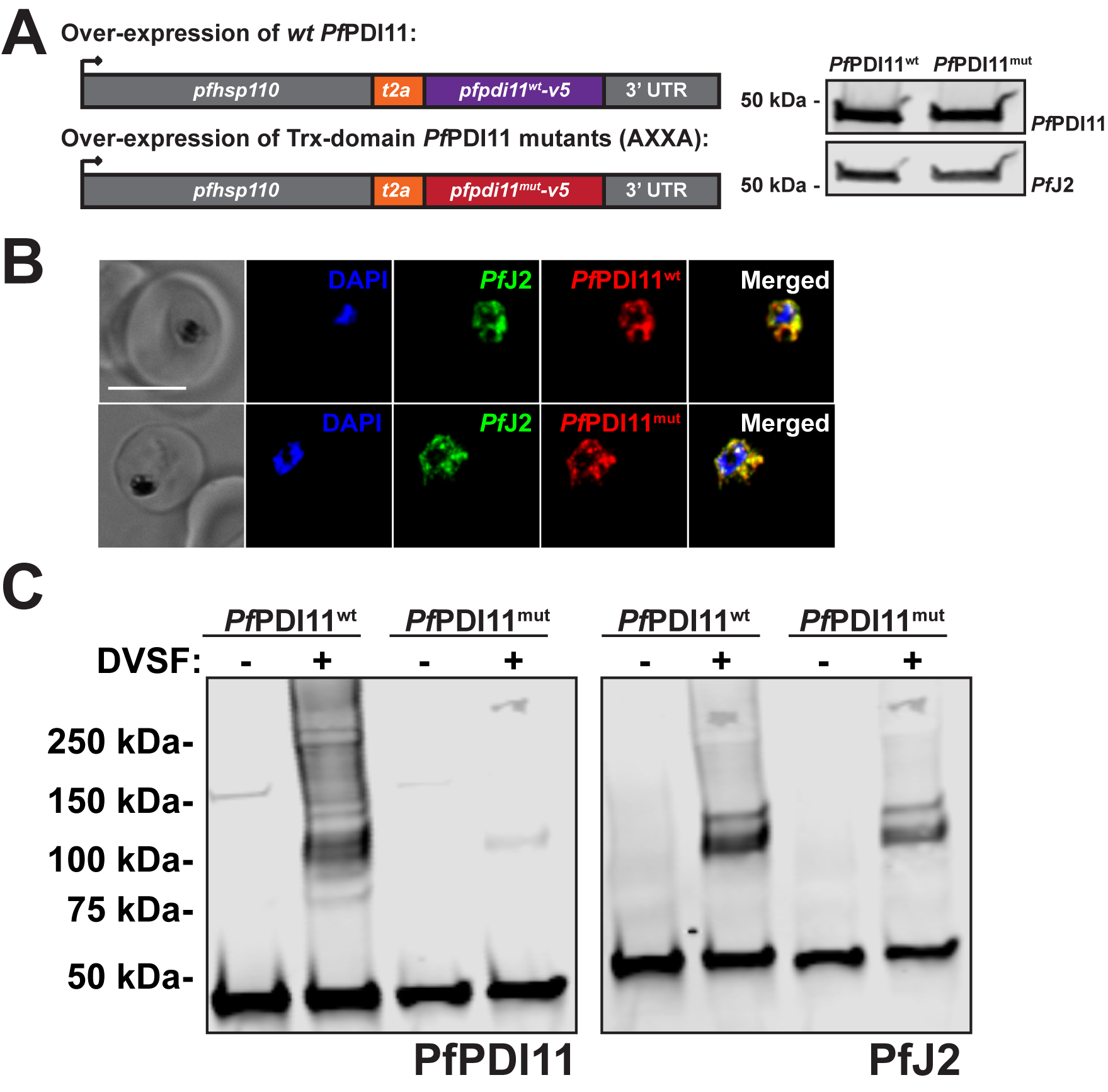

Supplement: S5 Fig — A) Left: schematic of exogenous V5-tagged, PfPDI11 expression using the pfhsp110 (PF3D7_ 0708800) locus and a T2A skip peptide in the PfPDI11wt and PfPDI11mut parasite lines, created in the background of PfJ2apt parasites. In PfPDI11mut parasites, both Trx-domain CXXS active sites were changed to AXXA. Right: western blot of PfPDI11wt and PfPDI11mut parasite lysates, probed for antibodies against the V5 tag (PfPDI11) and the HA tag (PfJ2). B) PfPDI11wt and PfPDI11mut parasites were glutaraldehyde/paraformaldehyde fixed and used for IFA. Staining was carried out using DAPI (blue), antibodies against the HA tag (PfJ2, green), and the V5 tag (PfPDI8, red). Scale bar represents 5 μm. C) PfPDI11wt and PfPDI11mut parasites were treated with 3 mM DVSF in 1x PBS for 30 minutes at 37°C, then parasite lysates used for western blotting. Membranes were probed with antibodies against the V5 tag (PfPDI11) and the HA tag (PfJ2). (TIF) [file ppat.1009293.s009.tif]

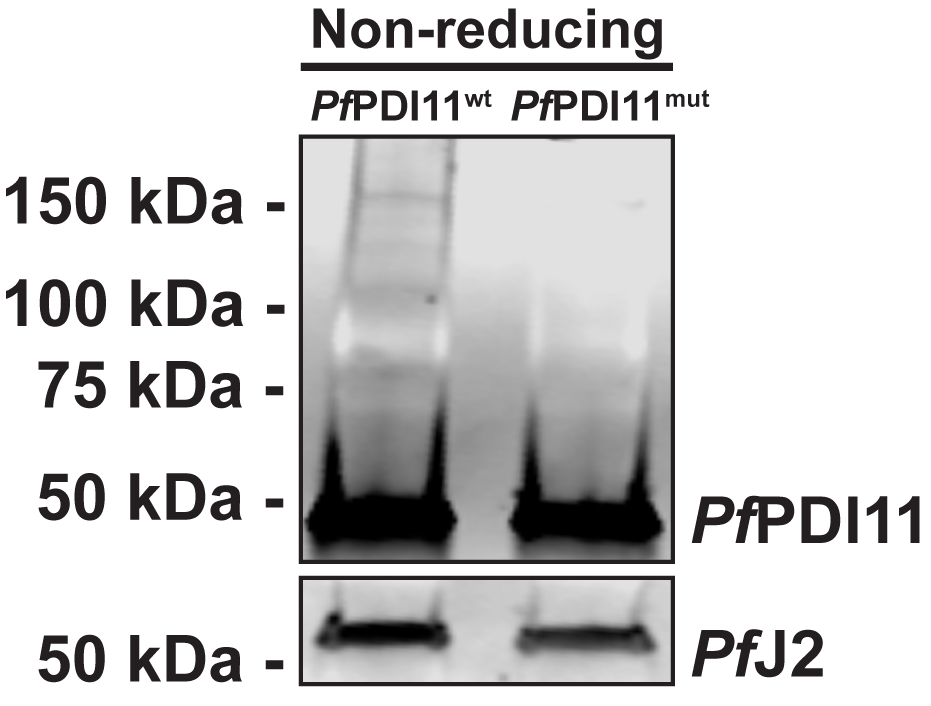

Supplement: S6 Fig — Saponin-isolated PfPDI11wt and PfPDI11mut parasites were dissolved in protein loading dye lacking a reducing agent and used for western blotting. Membranes were probed with antibodies against the V5 tag (PfPDI11) and the HA tag (PfJ2). (TIF) [file ppat.1009293.s010.tif]

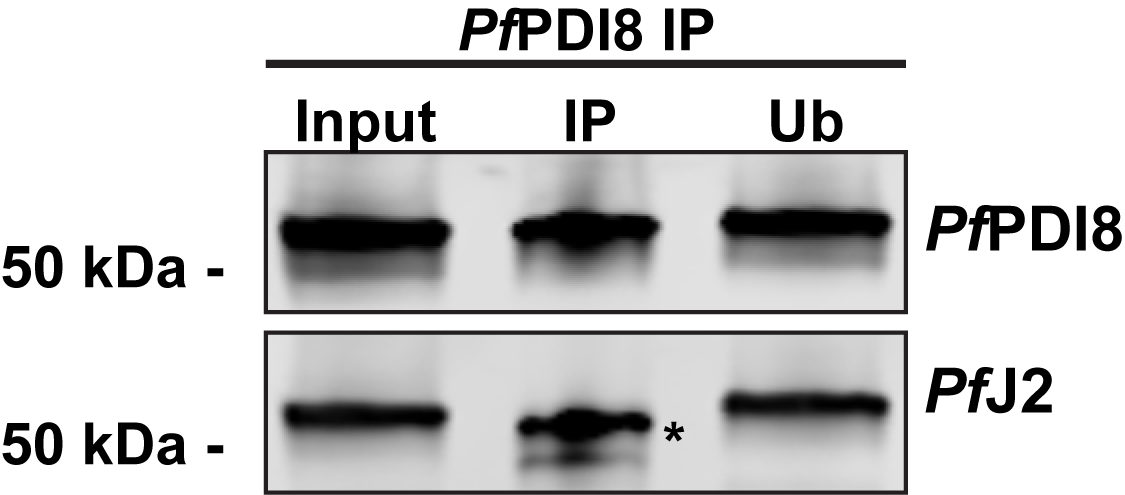

Supplement: S7 Fig — PfPDI8 and interacting proteins were immunoprecipitated from PfJ2apt-PDI8glmS parasite lysate using anti-V5 antibodies. The pre-IP input sample, the sample eluted from the antibodies (IP), and the sample removed from the beads containing the unbound proteins (Ub) were used for western blotting. Membranes were probed with antibodies against V5 and HA. The asterisk (*) denotes the heavy chain of the antibody used for immunoprecipitation. (TIF) [file ppat.1009293.s011.tif]

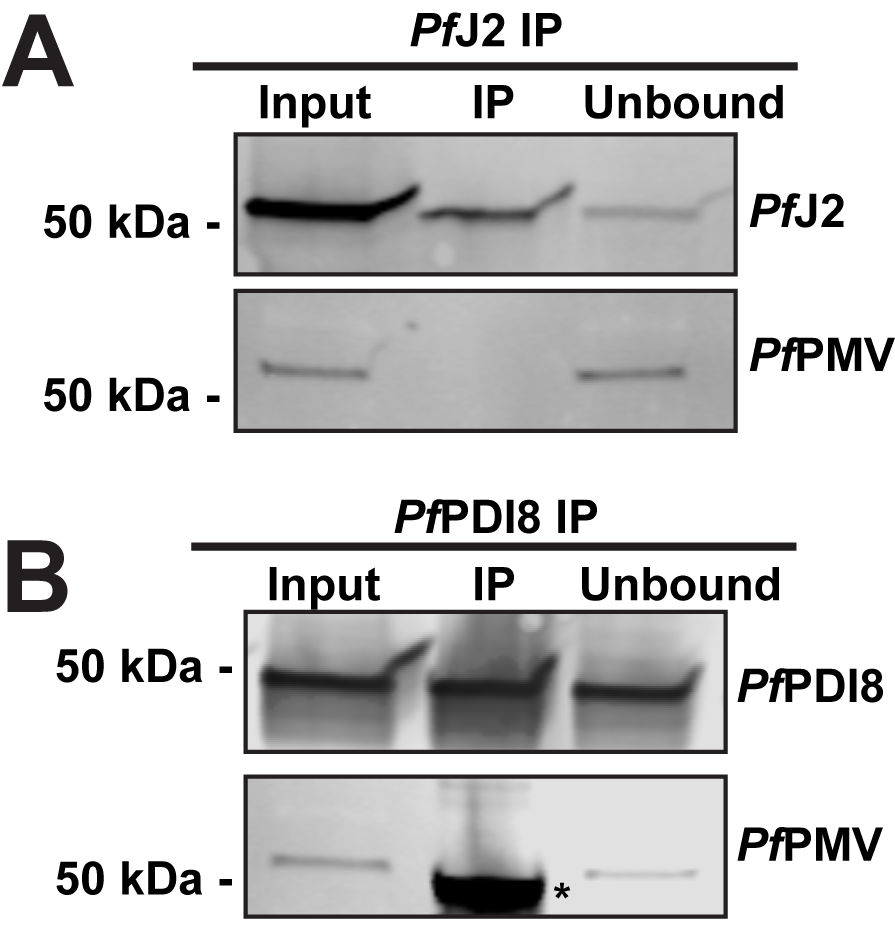

Supplement: S8 Fig — PfJ2apt-PDI8glmS parasite lysates were used for either (A) anti-HA immunoprecipitation (IP) or (B) anti-V5 IP. The pre-IP input sample (In), the sample eluted from the antibodies (Elu), and the sample removed from the beads containing the unbound proteins (Ub) were used for western blotting. Membranes were probed with antibodies against HA to detect PfJ2, V5 to detect PfPDI8, PfPMV. The asterisk in (B) indicates the heavy chain of the anti-V5 antibody used for immunoprecipitation. (TIF) [file ppat.1009293.s012.tif]

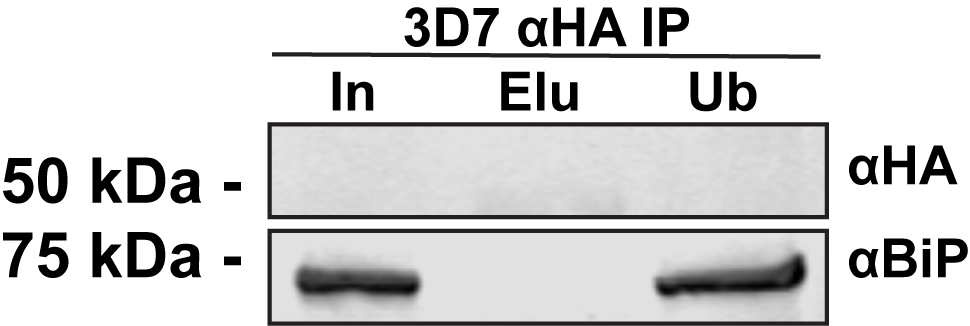

Supplement: S9 Fig — 3D7 parasite lysates were used for anti-HA immunoprecipitation (IP). The pre-IP input sample (In), the sample eluted from the antibodies (Elu), and the sample removed from the beads containing the unbound proteins (Ub) were used for western blotting. Membranes were probed with antibodies against HA and PfBiP. (TIF) [file ppat.1009293.s013.tif]

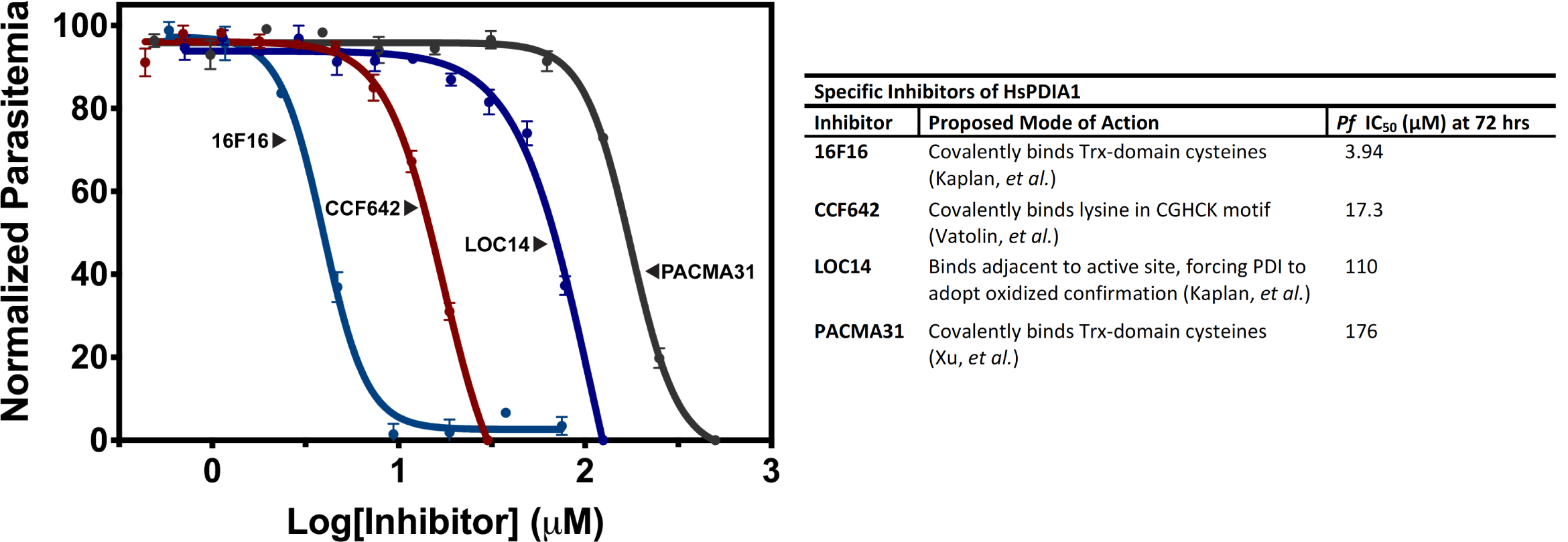

Supplement: S10 Fig — Left: Asynchronous 3D7 parasites were incubated in various concentrations of human PDI inhibitors. Each data point represents the mean parasitemia at a given concentration, in technical triplicate, at 72 hours. Error bars are not seen for data points in which they are smaller than the circle symbol, represent standard deviation from the mean. Representative IC50 curves are shown for each drug. Experiments were performed in biological triplicates. Right: table describing each of the human PDI inhibitors used and their calculated P. falciparum IC50 values. (TIF) [file ppat.1009293.s014.tif]
