# Supplementary material for: A redox-active crosslinker reveals an essential and inhibitable oxidative folding network in the endoplasmic reticulum of malaria parasites
Source: PLoS Pathog. 2021 Feb 3;17(2):e1009293. doi: 10.1371/journal.ppat.1009293 (PMC7886143; doi:10.1371/journal.ppat.1009293)
Supplement: Attachment — Submitted filename: 2020-10-28-PLoSPath-review-response.docx [file ppat.1009293.s015.docx]

As you will see, all 3 reviewers had a range of important concerns with the paper. All agreed that the functional significance of the diverse set of proteins identified in the pull-downs of PfJ2 was unclear, given the absence of further validation of many of the putative interactions, and that additional controls are essential for these experiments. Several related questions were raised over technical aspects of the immunoprecipitation experiments. Questions were also raised over the validity of DVSF as a selective, cysteine-reactive cross-linker (how well has its specificity been confirmed in previous work?) and the on-target selectivity of 16F16. Overall, there was a broad consensus that the conclusions of the work are overstated and not fully supported in some cases by the data. Citation of related previous work also requires attention.

RESPONSE: We have addressed the several specific concerns of the reviewers as noted below. The title has been changed in response to the reviewer suggestion. We included further validation and controls to bolster some our conclusions. The reviewer concerns were also addressed by toning down our conclusions in certain areas. We thank the reviewers for pointing out some citations we missed and these have been included in the revised manuscript.

Reviewer's Responses to Questions

**Part I - Summary**
Please use this section to discuss strengths/weaknesses of study, novelty/significance, general execution and scholarship.

Reviewer #1: Overall this is an interesting paper that characterizes several thioredoxin domain-containing proteins in P. falciparum. The experiments are interesting. However, the conclusions go significantly beyond the data in several places. Fundamentally it is a good paper but it should be written such that the conclusions are not overstated and are solidly supported by the data. If the authors are willing to do this it could be considered for PLOS pathogens. As the conclusions are currently overstated it is difficult to assess some aspects of the paper.

The authors initial focus is on the Trx domain-containing protein PfJ2. They make a tagged and conditional mutant of the PfJ2 encoding gene and show that the protein is ER-localised and important for normal blood stage replication. This is convincing.

RESPONSE: We thank the reviewer for recognizing the importance of this work. As the reviewer suggests, the conclusions have been edited to ensure that they are supported by the data.

The authors identify interacting partners of PfJ2 using immunoprecipitation. The authors provide a list of 23 proteins that are proposed to be specific interactors of PfJ2. The criteria for inclusion on the list includes presence of a signal sequence or TM domain and enrichment in the experiment over control. It is unclear how much confidence the reader should have in the majority of proteins on the list. If the signal sequence/TM criteria was not applied to the list, how many proteins would be similarly enriched in the experiment over control. The key question is do we have any confidence in the protein interactions that were not validated elsewhere. As the data is presented it is unclear how the reader should interpret this list - the data needs to be presented more clearly (also applies to S2).

The functional significance of this list of proteins is also unclear. The authors speculate that the list suggests that PfJ2 works in the ER with other chaperones and is important for folding of many proteins with diverse locations in the cell. This list of possible interactions does not really provide strong support this hypothesis. This would require a significant number of experiments to support this hypothesis; likely beyond the scope of a single paper.
RESPONSE: To address the reviewer’s concerns with the IP data, we have now included in the discussion the ways in which our mass spectroscopy IP data was subsequently validated for three of the proteins found to co-immunoprecipitate with PfJ2 (lines 906-912) and we have labeled explicitly in the MS data tables (Supplementary Table 1) the MS 1 intensity column that was used to identify proteins that were enriched in our PfJ2 IP experiments. We thank the reviewer for recognizing that validating every protein in the list is beyond the scope of a single paper. The data validate three of these interactions and in the case of two, we made double conditional mutants to test them further.

The authors then describe a series of experiments using the crosslinker DVSF. The cross linker appears to preferentially react with the highly reactive cys residues in redox proteins but it has not been widely used elsewhere (the three references provided appear to be the only references - if there are more the authors should provide them but this appears to be a relatively uncharacterised reagent). The experiments are interesting and provide interesting data but the section needs to be re-written with consideration of the following.
RESPONSE: We thank the reviewer for recognizing the importance of this reagent.

The authors use cross linking to support various points;

The first point demonstrated using DVSF is 'PfJ2 is redox active' - the protein does contain a Trx domain so likely contains reactive cys residues. Does the fact that it can be cross linked to other proteins mean that the protein is redox active. What exactly does 'redox active' mean when defined in this way? What is the significance of this observation and the statement that the protein is 'redox active'.
RESPONSE: We have also clarified our language into the proposed “redox active” roles we propose for PfJ2 and the PDIs by re-writing the Introduction to include what is known about Trx-domain proteins in P. falciparum (Lines 120-134), the specific proposed roles for Trx-domain proteins in the ER like PfJ2 (lines 136-173). We have also generally toned down our conclusions or included in the discussion a clearer justification for our conclusions.

The second point demonstrated using DVSF is 'We next sought to test the hypothesis that PfJ2 contributes to ER oxidative folding by determining whether its putative Trx domain is redox-active and capable of forming mixed disulfides with its clients. To this end we employed the bifunctional, electrophilic crosslinker divinyl sulfone (DVSF)...'

Crosslinking with DVSF indicates that two proteins can be cross linked with DVSF. It in no way shows that a protein is involved in oxidative folding or forms mixed disulfides. Likewise, it does not show whether a protein is a client of a thioredoxin domain protein or vice versa. If this is the conclusion that the authors wish to put forward they would need to do many more experiments to look at protein folding in the cell directly and assess the impact of mutants on the folding of a defined substrate.

RESPONSE: We disagree that DVSF crosslinking does not show that a protein can form mixed disulfide. If it cannot form a mixed disulfide then we will not get two proteins crosslinked because this is a bifunctional crosslinker that requires two cysteines to be within 7 Å and DVSF can form covalent bonds only if the two cysteines to act as nucleophiles. To address the specificity of DVSF, we expanded our discussion of the work done in yeast and mammalian models to validate the compound’s ability to specifically trap proteins undergoing disulfide bond exchange and how our own controls agree with that validation (lines 464-486, line 956-966).

The third point demonstrated using DVSF is: 'This specificity allows DVSF to covalently and irreversibly trap Trx-domain proteins to their redox substrates'
There is no indication that any of the cross linked proteins are substrates (as above). If anything, it seems likely that the cross linked proteins, such as PDI8, play a role in resolving the disulfides in PfJ2.
The experiments using DVSF are interesting but to demonstrate all of these points would take more data than could reasonably be crammed into one paper. The claims are quite bold and would require significantly more than cross linking with DVSF to demonstrate. However, the experiments do provide useful information. The authors need to state the aims and conclusions of each experiment in molecular terms that are clear, precise, accurate, and not overstated, and use these terms consistently throughout the paper. My major objection is the overstatement of the conclusions that are not supported by the data.
RESPONSE: The data show that DVSF does not crosslink other cysteine-containing ER proteins that are known to interact with several proteins, Plasmepsin V (16 cysteines; Figure 4A), and PfBiP (2 cysteines; Supplementary Figure 2). In the case of PDI11, if we mutate the active site cysteine, then it cannot crosslink, suggesting that DVSF needs a redox active cysteine. PDI11^mut^ has the active cysteine mutated but has 2 other intact cysteine residues but we do not observe any PDI11^mut^ crosslinking to other proteins (Supplementary Figure 5C). These data along with data from yeast and mammalian systems strongly suggest that DVSF is only able to crosslink cysteines, if they are redox-active.

The authors show that the PfJ2 can be cross linked using DVSF to several other proteins including PfPDI8 and 11. The experiments knocking down the genes that encode these proteins are interesting and seem convincing. The recruitment of BiP to the complex after cross linking could be discussed more critically in the discussion.

In the experiments using the 16F16 inhibitor of PDI it should also be mentioned that many other thioredoxin domain containing proteins could also be targets of the drug in addition to those studied by the authors.

RESPONSE: We agree and have modified the discussion as suggested by the reviewer (lines 826-828).

Other points:

Discussion - Generally, the conclusions are overstated. For example, the authors state 'Whether PfJ2 homologs in other eukaryotes recruit BiP in this fashion remains to be seen, but our data suggest that this role may be conserved in other eukaryotes'. Given that all of the experiments are done in plasmodium I don't really see how the authors come to this conclusion? There are other examples of overstated conclusions also.
RESPONSE: We have removed this from the revised submission. Given the conservation of BiP between all eukaryotes, we believe it is a reasonable speculation.

Table 1: Fold change - probably should be fold-enrichment.

RESPONSE: We have made this change.

Supplementary figure 7 - experiment aims to show formation of mixed disulfides. It would be preferable to show both the reduced and non-reduced gels.
RESPONSE: Other figures in the paper show reduced gels in the paper (Supp. Fig. 5C), therefore, we show the non-reduced gel here.

MS tables - data columns are formatted differently between experiments making comparison difficult.

REPONSE: The mass spectrometry experiments were performed at two different facilities, hence the formatting changes. The MS1 intensities in all reps are highlighted and the data are tabulated and graphed in Fig. 3.

Reviewer #2: This manuscript by Cobb et al describes work characterizing redox-active proteins of the P. falciparum ER.

The authors used the TetR-PfDOZI aptamer system to generate conditional mutants of the redox-active ER chaperone, PfJ2, and showed that it is essential for parasite survival. This is anticipated given its low mutagenesis score from the Zhang et al largescale piggyBac insertion mutagenesis study.

The authors determined the cellular location as the ER. This is consistent with a previous study which the authors have failed to cite (Oehring et al 2012; <https://genomebiology.biomedcentral.com/articles/10.1186/gb-2012-13-11-r108>)

RESPONSE: We have cited this study and apologize for this omission.

The authors used immunoprecipitation methods in an attempt to identify interacting proteins. The authors used a filter on the mass spec data. They state that they only considered proteins that were “present in all three PfJ2apt coIP experiments, and were at least 5-fold more abundant compared to the Controls” (i.e. no antibody). They employed an additional filter and only considered proteins containing a signal peptide and/or at least one transmembrane domain.

The IP identified a very large number of proteins which seems inconsistent with a successful pull-down of PfJ2 interacting proteins. That is, there appears to be a lot of non-specific precipitation, with many proteins that are known not to be ER-located ranked more highly in the numbers of peptides identified. Indeed, PDI-11 and PDI-8, proteins that the authors have singled out for further study, are ranked 1494th and 3350th in PfJ2 Rep 3 pull-down (Supp Table 1). It is also not clear exactly how the “5-fold more abundant” filter was applied. For example, the bait protein is identified as the 10th hit in the PfJ2 Rep 1 (with 33 peptides) and as the 38th ranked hit in the Control Rep 1 (with 28 peptides). This confusion may just arise from inadequately described methods and sub-optimal data presentation. As another example of issues with trying to assess the data in Supp Table I, the third replicate (PfJ2 Rep 3) is presented in a different format to the other two replicates.

One of the proteins listed in Table 1 is PF3D7_1324900 - L-lactate dehydrogenase. One would anticipate that this glycolytic enzyme would be located in the parasite cytoplasm.

RESPONSE: The reviewer is correct that most IP experiments, especially, chaperone IPs have many non-specific interactions. Therefore, we used a parental control IP as our comparison group. The number of peptides can be misleading because those depend upon the protease used to generate the peptides, size of the protein, protein abundance, and upon the ability of individual peptides to ‘fly’ in the mass spectrometer, which is controlled by peptide composition. Therefore, using the number of peptides can be misleading. We and others (Florentin et al 2020 PMID: 31482878 and Boucher et al 2018 PMID: 30212465) have used the MS1 intensities (highlighted in yellow, in the supplementary table 1) to estimate the abundance because this can be normalized, within a run, to the total MS1 intensities of ALL peptides within a run. These normalized MS1 intensities therefore, allows us to compare the PfJ2 IP with the control IP. This provides a better estimate of the fold-enrichment over the control, which means that abundant proteins will only appear in they are specific interactors. We also moved figures from supplementary files into the main-text of the paper (Figure 3) that better demonstrates how we chose proteins as high-confidence PfJ2 interactors. At the reviewer’s suggestion, we also generated a new figure (Supplementary Figure 8, lines 702-713) to show that our anti-HA and anti-V5 IPs are specific.

We agree that L-lactate dehydrogenase (PF3D7_1324900) would be in the cytoplasm and to exclude these proteins our data was filtered to remove all proteins without a signal peptide or at least 1 transmembrane domain from further analysis and are not present in the new Figure 3. The MS1 intensities of these proteins were, however, used to calculate the total MS1 intensities within a run.

The authors used an innovative redox-active cysteine crosslinker to identify its substrates, which are other mediators of folding in the oxidizing environment of the ER.

RESPONSE: We are glad that the reviewer appreciates the utility of the crosslinker.

The authors show, using the GlmS system, that knockdown of PDI-8 is associated with inhibition of growth while knock-down of PDI-8 is not deleterious. As the authors note, these studies are consistent with the relative mutagenesis score from the Zhang et al largescale piggyBac insertion mutagenesis study.

The authors were unable to generate transgenic parasite lines overexpressing PfJ2 or PDI-8. By contrast, a previous study (Oehring et al 2012) did report did generation of transgenic parasite lines expressing full-length C-terminally tagged PfJ2 from an episomally maintained plasmid. The authors should comment.
RESPONSE: While we acknowledge that Oehring et al were able to overexpress PfJ2 from an episomal plasmid (line 129), their use of an episomal plasmid and our attempt to integrate a copy of PfJ2 into the Hsp110 locus are two entirely different methods of overexpression. Therefore, we can’t directly compare their success to our failure to generate these specific transgenic parasites.

Co-IP experiments were used to confirm the interactions (Fig 6). It would have been useful to include as a control a protein that is not expected to be immunoprecipitated (eg a cytoplasmic protein).

RESPONSE: We agree, and included a control protein (Plasmepsin V, an ER protein) in the IPs (Suppl. Fig. 8).

The authors examined four commercially available PDI inhibitors. Only the irreversible alkylating agent, 16F16, showed any activity; and then only at uM levels. At this concentration, 16F16 is likely to react with thiol groups in multiple proteins. The authors could determine whether the PDI knock-down strains are more susceptible to 16F16 as a means of determining its activity is on-target. Without such validation it is over ambitious to claim that PDIs are a plausible target for new antimalarials.

RESPONSE: We agree and have edited lines 826-828 to include the suggestion that 16F16 likely inhibits several Trx-domain containing proteins. The data do suggest that PDIs activity is inhibited by 16F16 and because it inhibits the activity of two essential Trx-domain proteins (and possibly more). Therefore, since 16F16 has multiple targets, knocking down one protein to see enhanced susceptibility does not work. We disagree that PDIs are not valid targets for new antimalarials because the data does show that 16F16 inhibits protein activity. While we agree that 16F16 is not good antimalarial lead, these data do suggest that more potent compounds may be generated, though that is beyond the scope of this paper.

Minor points:

Line 288 - The authors state “.. PfPDI8 and -11 remain unstudied in vivo..”. It would be better to say “remain unstudied in cultured parasites”. “in vivo” implies that the studies involve infected animals.

RESPONSE: We have changed our use of “in vivo” to “in cultured parasites” at the reviewer’s suggestion.

Fig 2D. The images of the untreated culture suggest that the line has a very short life cycle. Rings are observed at the same stage a 0 h and 39 h. Is this a particular trait of this 3D7 line?

RESPONSE: Yes, this 3D7 line has an accelerated intraerythrocytic life cycle of about 40 hours.

Reviewer #3: The manuscript “A druggable oxidative folding pathway in the endoplasmic reticulum of human malaria parasites” describes interesting and important work that merits publication pending major modifications.

In this work, Cobb and co-workers explore the role of ER oxidative folding in malaria. This in presented in context of a new avenue for drug discovery. Within this discussion, it would be courteous of the authors to mention the pre-existing literature referring to redox regulation of protein functions as potential drug target for apicomplexan parasites, which includes (but not limited to) work by the Becker and Muller groups in the malaria research field, and publications on the apicomplexns Toxoplasma gondii and Eimeria tenella.
The authors select an ER protein with Trx domain (no explanation provided for this selection, but should be) to start with. Genetic knockdown shows essentiality and microscopy is used to validate its ER location (though the section about trafficking signals is hard to follow for non-experts). IP experiments are used to discover putative partners and proposed clients, however an essential control is missing for this part (see below).
DVSF is used to demonstrate redox activity, however since this was not used before a validation is needed (see below). Co-IP post DVSF treatment is used to again capture putative redox interactors, focus on PDI8 and 11, and knock down demonstrated that PDI11 is essential. Reciprocal co-IP was used in attempt to validate J2/PDI8/BiP interactions however requires controls (see below). Finally, drugs discovered as human PDI inhibitors were shown to be toxic to Plasmodium and compound 16F16 reversed the observed DVSF cross-linking of J2, PDI8 and PDI11, leading to an extrapolation (see below) that redox partnerships are a good target for development of anti-malaria drugs.

RESPONSE: We apologize for failing to cite the Trx-domain protein work more broadly and have corrected this (lines 99-113), and we have explicitly stated our rationale for choosing to work on Trx domain proteins in the ER (lines 115-136). We have also provided more details regarding the validity and specificity for using DVSF in the manner that we did and how our experiments and controls align with previous validation (lines 464-486, line 956-966). In particular, the reviewer requested a western blot probing for a control protein which contains cysteines that are not involved in disulfide bond exchange and is therefore not crosslinked by DVSF; we agree that this is a foundational control and the data are presented in Figure 4A and lines 457-460, as well as Supplementary Figure 2. At the reviewer’s suggestion, we have also included control western blots (Supplementary Figure 8, lines 702-713) probing for PfPMV to show that our anti-HA and anti-V5 immunoprecipitations are specific and the observed interactions are not non-specific.

Reviewer #3: Major issues:

DVSF treatment is used to demonstrate redox activity and redox mediate interatcions, however since this method is used in the ER and in Plasmodium for the first time, it requires validation. How do we know that DVSF doesn’t simple link together any cysteine containing proteins in the very small ER of these organisms? A control whereby an ER protein with a cysteine known/expected no to be involved in disulfide exchange is necessary.
RESPONSE: We agree that this is a critical control to show DVSF does not crosslink any protein with cysteines. The data are presented in Figure 4A and lines 457-460 and show that DVSF does not crosslink Plasmepsin V (16 cysteines) or BiP (2 cysteines, Supplementary Figure 2), which are ER proteins that interact with several proteins that transit the ER.

The identification of putative partners and clients via co-IP lacks rigor and a demonstration of functional relevance. The co-IP results described in line 179-198 find a broad range of ER proteins many of which may well be false positive. An appropriate control is to compare to a co-IP experiment with an unrelated tagged ER protein that is not expected to interact with the suggested partners or clients. PfMV would be prime candidate for such control according to Figure 3A.
Some clients found in said co-IP are highlighted in the result section and an extrapolation on the role of oxidative folding in their function is included in the discussion. This should be demonstrated. For example, the authors mentioned MSP1. A reciprocal co-IP to validate this proposed interaction should be included. And/or the authors can test if the J2 knock-down prevents function or biogenesis of MSP1. This is just an example. Some functional work is necessary for at least one proposed client.

RESPONSE: At the reviewer’s suggestion, we have also included control western blots (Supplementary Figure 8, lines 702-713) probing for PfPMV to show that the anti-HA and anti-V5 immunoprecipitations are specific and the observed interactions are not non-specific. Three potential interactors are confirmed (BiP, PDI8, and PDI11) and validating every interaction would be beyond the scope of this paper. The PfJ2 knockdown results in parasites dying before MSP1 is processed and trafficked to the merozoite membrane, so it is not possible to observe biogenesis of MSP1.

The proposed interaction between J2/PDI8/BiP is not well supported. All the co-IPs shown in Figure 6 do not show the wash and there is no control to show that the IP material doesn’t contain unspecific interactors. To demonstrate co-IP that we should see that the protein of interest isn’t simply a contaminant of all fractions including wash, and that irrelevant proteins don’t contaminate the elution. In addition, even if evidence for co-IP upon DVSF treatment is provided, this still suffers from lack of control of whether the proteins that DVSF link together are true interaction partners in physiological conditions. Finally, the data obtained with DVSF is not consistent with the proposed interaction with BIP.

RESPONSE: The proposed controls are included and we show that a non-specific ER protein like PMV do not co-IP and show the unbound fractions as well (Supplementary Figures 8 and 9). The data show that DVSF crosslinking is specific and therefore, suggest that these are true interaction partners under physiological conditions (Figure 4A, see response to Issue 1). We are unsure what is meant by ‘data obtained with DVSF is not consistent…… interaction with BiP’. BiP is does not crosslink to anything in the presence of DVSF (Supplementary Figure 2), this is consistent with the fact that neither of the two cysteines in BiP are redox-active.

The proposed interference of drugs with redox interactions also lack rigor. Figure 7 B,C and D – it is important to show a protein stain of same gel run of treated parasites to conclude that it is a specific effect of blocking DVSF mediated cross-linking. Other measures of parasite biology should be included to conclude specific effect on ER oxidative folding as opposed to other defects. Even if those aspects are satisfied, the discussion of drug discovery potential should be less biased and highlight also caveats: disulfide exchange is a highly conserved mechanism posing a challenge to obtain specificity to one organism over another. What is known about the compounds selected, their toxicity in animal models, their potential therapeutic window etc etc
To enhance the functional aspect – assuming a client is validated as suggested above, interaction with said client should be disrupted by 16F16, which would provide further support to the proposed inhibition mechanism.

RESPONSE: We have changed the title as suggested by the reviewer and lowered our focus on the drugging of these redox activities. We agree, the data only suggest that these redox processes can be blocked but not that 16F16 is specific only for these redox proteins, our modified conclusions make that distinction (lines 826-828). The quantification of DVSF-blocking activity of 16F16 is done by using a ratio of crosslinked vs non-crosslinked protein. If there were issues with loading, as the reviewer correctly worries, then this would have an equal effect on both sets of highlighted bands (Figure 8), and therefore, would not cause a difference in the ratio of the two highlighted bands. We have also included more discussion on the ability to target such conserved biological mechanisms. We are thankful to the reviewer for pointing out that there are several challenges in using these compounds as antimalarials. We agree that there is a lot of work to be done before we can propose the use of these compounds as antimalarials because 16F16 itself is unlikely to be an antimalarial and we do not propose to using this compound as one. The data show that 16F16 inhibits the redox activity of three Trx-domain proteins in the ER, two of which are clearly essential for the asexual cycle. However, as we discuss, there is a long road ahead of us from identifying a potential target to actually developing a usable antimalarial. As the reviewer notes, there are several issues to be resolved such as animal toxicity, therapeutic windows etc. This would require a medicinal chemistry program coupled to these redox assays and would be well beyond the scope of this paper.

**Part Part II – Major Issues: Key Experiments Required for Acceptance**
Please use this section to detail the key new experiments or modifications of existing experiments that should be absolutely required to validate study conclusions.

Generally, there should be no more than 3 such required experiments or major modifications for a "Major Revision" recommendation. If more than 3 experiments are necessary to validate the study conclusions, then you are encouraged to recommend "Reject".

Reviewer #1: As it is, the conclusions go so far beyond the data that it is difficult to make suggestions of experiments. Likely, rewriting with significantly less speculative conclusions would make it easier to assess the paper.

RESPONSE: We have made these changes.

Reviewer #2: More convincing data for the IP experiments (Supp Table I) is required including an example of how the "5-fold more abundant filter was applied.
RESPONSE: Figure 3 now shows how the filter was applied

Additional control are needed for the co-IP experiments (Fig 6).
RESPONSE: We have included additional controls (Supplementary Figures 8 and 9, lines 702-713) to demonstrate the specificity of the IP.

Further evidence that 16F16 is on target is needed.

RESPONSE: We have modified our conclusions and included more discussion on 16F16. The data suggest that 16F16 probably inhibits the activity of several Trx-domain containing proteins, including PfJ2, PDI8, and PDI11.

**III – Minor Issues: Editorial and Data Presentation Modifications**
Please use this section for editorial suggestions as well as relatively minor modifications of existing data that would enhance clarity.

Reviewer #1: (No Response)

Reviewer #2: (No Response)

Reviewer #3: Minor issues

- title focuses on “druggable” process, but only the last figure deals with drugs and needs confirmation (see above) this title is misleading

RESPONSE: We have also changed the title of the paper to reflect the fact that “druggability” of Trx-domain proteins is not the sole focus of the manuscript.

- the example of how Trx domains and PDIs work in general should go in the introduction
- pfMV used as ER marker – provide a reference for this
- the introduction should include more information about what was previously known: about redox regulation in apicomplexans, about J2, about the compounds used in Fig 7

RESPONSE: We apologize for failing to cite the Trx-domain protein work more broadly and have corrected this (lines 99-113), and we have explicitly stated our rationale for choosing to work on Trx domain proteins in the ER (lines 115-136).

- EC50 of aTc used as measure for inhibition of growth – is that the acceptable way? Do other Plasmodium research group that use this DOZI system measure growth in this way? If so, provide reference.

RESPONSE: This is just one reasonable measure we use to show growth dependence on the presence of aTc. We use this as well as other measures to measure aTc dependence of the PfJ2 conditional mutants. All it shows is that the parasite growth is dose-dependent upon aTc presence.

- hits from co-IP are prioritised based on presence of SP or 1TMD. This doesn't provide any certainty of ER localization or transit other compartments (mitochondria, peroxisome, plasma membrane) can be targeted this way without passing through the ER

RESPONSE: We agree and SP or 1TMD is just used as a filter because proteins without these signals almost certainly do not transit through the ER. This is not to suggest that these proteins transit or localize through the ER, though the ones with a SP almost certainly do. We are not aware of mechanisms in *Plasmodium* parasites that allow for direct insertion of TMD-containing proteins directly into the plasma membrane without first transiting through the ER.

- provide explanation to why PfPD11 was not identified in the first coIP but was trapped with DVSF?

RESPONSE: We explain the presence of PDI11 in the DVSF crosslinking data but its seeming absence in the non-crosslinking IP data (lines 885-891). We did identify PDI11 in our non-crosslinked IP data but it did not meet our strict criteria for generating the list of proteins in Figure 3, which suggests that our criteria is strict enough to identify true interactors.

– In Figure 3C the first protein band of lane 2 is not clear.
- CXXS is referred to as active site throughout the manuscript, however it is not a Trx active site.

RESPONSE: The literature is not clear whether or not this is a Trx-active site. We refer to it as the active site because the data show that the cysteine in this motif does form mixed disulfides with other proteins (Figure 6B and Supplementary Figure 5, 6).

- since the downregulation in FigS4 isn’t complete it is not supported that PDI11 is not essential

RESPONSE: We agree, therefore, we state that ‘suggest that *Pf*PDI11 may be dispensable, though in the case of *Pf*PDI11, the lack of a phenotype could be attributed to incomplete knockdown’ (Lines 604-605).

- figure 7B,C,D – it should be marked on the gels what bands were measured for making the graphs, all the gels included for statistics should be provided as supplementary.

- Supplementary figure 10 and Figure 7. It would be beneficial to include the stated IC50 values of the drugs against human cells for comparison, even though this is a proof of principle experiment

RESPONSE: We also modified Figure 8 (previously Fig. 7) to clarify the areas of the western blots that we quantified for “cross-linked signal” and “non-crosslinked signal” and created a new Supplementary Table 3 to provide the quantification for all western blots across replicates.
