# Supplementary material for: A redox-active crosslinker reveals an essential and inhibitable oxidative folding network in the endoplasmic reticulum of malaria parasites
Source: PLoS Pathog. 2021 Feb 3;17(2):e1009293. doi: 10.1371/journal.ppat.1009293 (PMC7886143; doi:10.1371/journal.ppat.1009293)
Supplement: Attachment — Submitted filename: 2020-12-15-PLoSPath-review-response.docx [file ppat.1009293.s016.docx]

As you will see, two reviewers have raised minor points that require attention prior to the manuscript being considered further. Reviewer #1 requests that further clarification is provided regarding the criteria used to include proteins in the list of candidate partners identified in Figure 3. Could you please provide that clarification. Reviewer #3 requests that the title of the manuscript should be again modified in order to better describe its focus on characterisation of the PfJ2 redox network, rather than its potential as a drug target (which is indeed a relatively minor part of the work in terms of the number of figures devoted to it). While we believe this is a relatively less important issue than the first, we would still be grateful if you could give the point some consideration.

RESPONSE: The points raised by the reviewer have been addressed below. We have provided the clarification requested by reviewer 1 and we have modified the title of the manuscript to: ‘A redox-active crosslinker reveals an essential and inhibitable oxidative folding network in the endoplasmic reticulum of malaria parasites’. We hope that the revised manuscript is acceptable for publication. Thank you.

Reviewer Comments (if any, and for reference):

Reviewer's Responses to Questions

**Part I - Summary**
Please use this section to discuss strengths/weaknesses of study, novelty/significance, general execution and scholarship.

Reviewer #1: Overall the paper is interesting and contributes significantly to our understanding of Trx proteins in the parasite. Whilst there are inevitably some aspects that might be followed up this is probably beyond the scope of the paper.

Figure 3 (list of proteins identified in pulldown) - the description in the main text remains problematic.
The overall significance of the pulldown in figure 3 remains unclear simply because the criteria used to select the proteins containing a signal sequence or TM domain strongly biases the list. It is unclear whether this data could really be interpreted if this criteria was not used i.e. without excluding the non-ss and non-TM proteins how many proteins would be enriched over the control.
Filtering the data in this way does not provide a high confidence list of proteins. As it is, it cannot really be interpreted by the reader (the reader cannot really tell if 5 ER proteins were enriched in the pulldown but also 100 cytoplasmic proteins). This need to be clarified.

RESPONSE: We thank the reviewer for appreciating the importance of our study. As requested, we have added more description on how the criteria were used to select the proteins. While we agree that filtering based on signal sequence or TM does bias the list, this bias is biologically relevant. When we lyse the cell for the IP, cytosolic proteins will interact with PfJ2, however, these interactions are not biologically relevant because the ER membrane always separates these proteins within the parasite cell. We have run the analysis as requested, and as expected, we find numerous cytosolic proteins that are enriched over the control. This is not surprising because these are more abundant (like the ribosome) and PfJ2 has conserved sites that can interact with any Hsp70 (including the cytosolic ones), associated chaperones, ribosome, proteasome, and unfolded proteins. Chaperones are functionally promiscuous interactors, and cell lysis allows for interactions that normally do not occur *in situ* because of compartmentalization. For example, cytoplasmic proteins in this list are proteins like the cytosolic Hsp70 (70-fold enriched), the cytosolic Hsp110 (~40 fold enriched), several ribosomal proteins, and several 20S proteasome complex proteins. However, these interactions are not biologically relevant as these proteins are not in the same cellular compartment as PfJ2. Of note, our previous work another ER chaperone did not identify the same proteins as in this filtered list, suggesting that our approach is identifying biologically relevant interactors (Kudyba et al 2019, PMID:31087747). Therefore, we have changed the results section to clarify this and we term the SP/TM filtered list as ‘biologically relevant interactors’ instead of high-confidence interactors (lines 177-180). We added more information in the methods section as well to clarify how this list was generated (lines 703-706).

Reviewer #2: The authors have addressed my queries and suggestions.

RESPONSE: We are pleased to note that we have addressed all reviewer queries.

Reviewer #3: The revised manuscript now provides the critical controls for the main strategy used. Likewise the discussion in the context of drug discovery is revised. This satisfies my concerns.

RESPONSE: We thank the reviewer again for suggesting important controls that strengthens the conclusions of our study.
